# Evaluation of Self-Perceived Body Image in Adolescents with Mild Idiopathic Scoliosis

**DOI:** 10.3390/ejihpe12030023

**Published:** 2022-03-11

**Authors:** Guido Belli, Stefania Toselli, Pasqualino Maietta Latessa, Mario Mauro

**Affiliations:** 1 Department of Sciences for Life Quality Studies, University of Bologna, 47921 Rimini, Italy; guido.belli@unibo.it (G.B.); pasqualino.maietta@unibo.it (P.M.L.); mario.mauro4@unibo.it (M.M.); 2 Department of Biomedical and Neuromotor Sciences, University of Bologna, 40126 Bologna, Italy

**Keywords:** body image, AIS, QoL, health, SRS-22

## Abstract

Adolescent idiopathic scoliosis (AIS) is the most prevalent types of scoliosis, affecting up to 3% of children around the world. The progression of AIS can cause alteration in psychological components such as self-perceived body image and self-identity, which negatively affect the teenager quality of life (QoL). The mainly aim of this cross-sectional study is to investigate how mild AIS impacts self-perceived body image in young people. Fifteen participants (mean age = 14.47 ± 2.825) of both sexes (male = 5; female = 10) with a curve magnitude from 10° up to 25° completed the Scoliosis Research Society Patient Questionnaire (SRS-22), the Trunk Appearance Perception Scale (TAPS) and were subject to spinal analysis and photogrammetry. Results display statistical differences between self-perceived body image and other SRS-22 domains (*Hotelling t*^2^= 70.29; *F*_(3,12)_ = 20.08; *p* < 0.001). Additionally, the regression model, which better explained the self-perceived variability, was fit by function/activity, pain, and mental health domains (*F*_(4,10)_ = 4.39; *p* = 0.029; *R*^2^ = 0.545). Although AIS was not severe, it negatively affected participants self-perceived body image. More attention in AIS qualify of life is needed, and early treatments could be necessary to prevent psychological impairments self-perception related.

## 1. Introduction

Scoliosis is a three-dimensional complex deformity of the spine mainly characteristic in the frontal plane [1]. In the current literature, several types of scoliosis are described, and the idiopathic form in adolescence (Adolescent idiopathic scoliosis: AIS) is the most prevalent, representing 80% of total cases [2,3]. AIS affects up to 2–3% of children around the world, and its occurrence is more likely in girls [4]. AIS is characterized by a radiological spinal curve of at least 10° in the frontal plane (measured by the Cobb method), with different grades of vertebral axial rotation [1]. Although radiographic image is the current gold standard for AIS diagnosis, several non-invasive devices and surface metrics have been validated to measure spinal curvatures and trunk shape in the last years [5,6,7]. In particular, SpinalMouse^®^ and photogrammetry have been proposed like non-invasive and reliable methods for spinal analysis and postural evaluation in AIS [8,9]. Surgical and non-surgical interventions have been compared to manage prognosis in people with AIS and different treatments are recommended to improve physiological, functional, and psychological conditions [1,10,11].

Even though AIS rarely causes any health problems during growth, the scoliosis progression can lead to a visible trunk deformity and elicit psychological disturbances [5,12]. Specifically, self-perceived body image and self-identity could be negatively affected and consequently reduce the AIS people Quality of Life (QoL) [13]. For example, a recent review showed that participants with AIS reported dissatisfaction with physical appearance, which could lead to feelings of “embarrassment” and “inferiority” [14]. Although approximately 40% of youth with AIS experienced some alterations in QoL, it is still unclear which are the most involved aspects [15]. In recent years, an increased interest has been focused on self-perceived body image and some specific assessment tools have been developed. Among all, the Scoliosis Research Society Patient Questionnaire (SRS-22) and the Trunk Appearance Perception Scale (TAPS) are commonly used by clinicians and therapist during AIS evaluation [16,17]. SRS-22 is a five-domain questionnaire that consists of 22 items regarding function/activity, pain, self-perceived body image, mental health, and satisfaction of treatment. TAPS is a visual scale that allows evaluating self-perception of the trunk and deformity in scoliosis by three sets of body figures. However, previous studies reported different viewpoints on linear correlation between spinal curve magnitude and body image self-perception [18,19,20]. Furthermore, whether mild scoliosis could impact AIS people self-perceived body image is still debated [21,22,23].

The main purpose of the present study is to investigate whether the mild magnitude of the curve affects self-perceived body image measured by SRS-22 in people with diagnosis of AIS. In addition, we will compare SRS-22 self-image domain with others in order to understand the relation with different aspect of QoL. Finally, we will perform several regression models to analyze whether the self-perceived body image can be explained by the QoL, TAPS, SpinalMouse^®^, and photogrammetry measurements.

## 2. Materials and Methods

### 2.1. Design and Participants

This is a cross-sectional study design. Participants were recruited from Fisiokinè Medical Centre (Scandiano, Reggio Emilia). The criteria of selection included a diagnosis of adolescent idiopathic scoliosis, no other morphological alterations, no prior surgical intervention for scoliosis, magnitude of the main curve ranging from 10° up to 25°, and age from 12 to 22 years old. No gender restrictions were defined. All participants were informed and gave voluntary consent to participate in the study. A parents’ consent was requested when a participant was younger than 18 years old. Privacy criteria were met. The study was approved by the Bioethics Committee of the University of Bologna and was conducted in accordance with the guidelines of the Declaration of Helsinki; project identification code was n.2.18. Project data, instruments and analysis script have been shared via OSF.io (https://osf.io/wcq2h/?view_only=a7c16c3f100040cf86ce00e70a6c03d7, accessed on 28 February 2022).

During the recruitment phase, each participant completed the anamnesis investigation. All specific medical reports were collected and analyzed to meet selection criteria. Recruitment phase lasted two months, from November to December 2020.

### 2.2. Measurement Instruments

#### 2.2.1. Scoliosis Research Society Patient Questionnaire (SRS-22)

The SRS-22 is a 22-items questionnaire composed of five domains: function/activity, five items; pain, five items; self-perceived body image, five items; mental health, five items; satisfaction with treatments, two items. Each item is scored form one (worst) to five (best). A previous study reported high values of the internal SRS-22 consistency (0.75 < α < 0.92; α, Cronbach’s alpha) and intraclass correlation coefficient (0.85 < ICC < 0.96) [24].

In the present study, an Italian SRS-22 version was used [16]. The results are expressed as the mean value for each domain, calculated as the total sum of the domain divided by the number of items, and the total score. The domain five was no used because participants did not receive any physical treatment. Additionally, in order to analyze the differences between domain three (self-perceived body image) and other domains (one, two and four), the total mean with no domains three and five was calculated.

#### 2.2.2. Trunk Appearance Perception Scale (TAPS)

The TAPS is perception scale which evaluates self-perception of trunk appearance and deformity in scoliosis. It includes three sets of figures that depict the trunk from three viewpoints: set one, looking toward the back; set two, looking toward the head with the patient bending over (Adam’s test); set three, looking toward the front. The last view has two sets of drawing, detected for each gender. Participants must choose only one figure for each set, which represents its self-perception. Each drawing is scored from one (greater deformity) to five (smallest deformity). The final score is calculated as a mean of all sets. A previous study reported a Cronbach’s alpha value of 0.89, and an intraclass correlation coefficient of 0.92 [25].

#### 2.2.3. Spine Analysis

In order to evaluate spinal curves and trunk alignment, the SpinalMouse^®^ (IDIAG M360^®^, Fehraltorf, Switzerland) was used. It is a non-invasive computer-assisted medical device that quantifies the curvature and mobility of the spinal column in the frontal and sagittal planes, by gliding it manually along the spine [26,27]. Data are sampled every 1.3 mm while the mouse is rolled from vertebra C7 to S3, giving a sampling frequency of approximately 150 Hz. Results are wirelessly transferred to a computer, where the IDIAG software displays vertebral positions, joint angles and spinal. A recent study reported a high correlation between Cobb angle evaluated with X-ray and intra (ICC = 0.872) or inter-observer (ICC = 0.962) SpinalMouse^®^ measurements [8].

**Figure 1:**
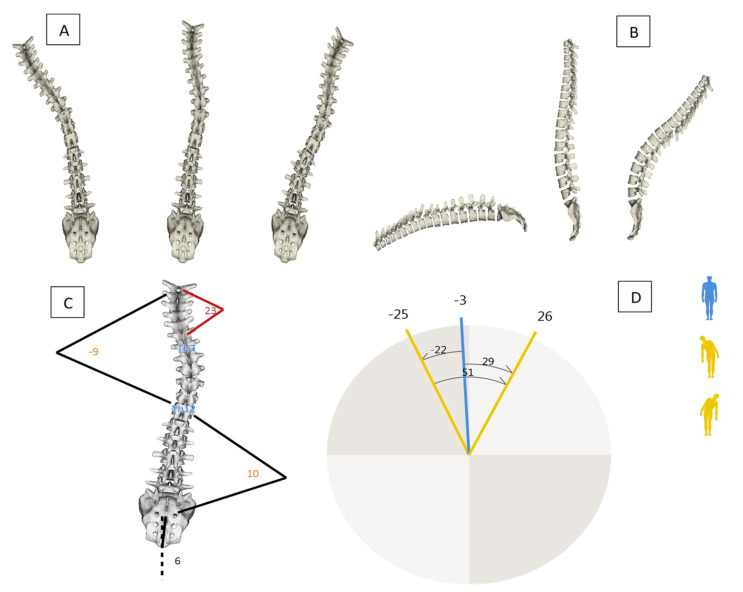
Examples of SpinalMouse measurements: (**A**) spine morphology on frontal plane; (**B**) spine morphology on sagittal plane; (**C**) curves amplitude on frontal plane; and (**D**) trunk inclination on frontal plane.

In the present study, the SpinalMouse^®^ measurements were performed by a trained specialist with more than five years’ experience. Data were collected in a quiet and well-lit environment with a comfortable temperature. The evaluation was settled in the morning to avoid positional differences of the spine due to fatigue and/or daily stressful factors. After undressing the upper body, the C7–S3 vertebral spinal processes were determined and marked with a dermo graphic pen by the specialist while the patient was standing up in the anatomical position. Measurements were performed in 6 different trunk positions during standing: neutral, maximal flexion and extension, for sagittal plane evaluation; neutral, right and left lateral flexion, for frontal plane evaluation. In neutral position (both sagittal and frontal planes), participant was asked to maintain a relaxed position, looking, and facing horizontally toward the wall, with the feet shoulder width apart and straight knees and arms by the side. In maximal flexion, the subject was asked to flex the trunk with extended legs as far as possible, aiming to touch the ground with fingertips. In maximal extension, the participant was asked to cross arms in front of the chest and extend the trunk as far as possible, without extension of cervical spine. In lateral flexion (right and left), participant was asked to flex trunk laterally, with arm over leg side. SpinalMouse^®^ was then moved downwards along the spinal criteria points, in each position. Participants did not perform a warm-up before the examination. Only three measures were extracted and analyzed from all raw data available. The three variables were the inclination of spine on sagittal plane, the inclination of spine on frontal plane, and the thoracic and lumbar curvature on the frontal plane. Figure 1 shows some of all SpinalMouse possible measurements displayed by IDIAG M360 software and used in the present study.

#### 2.2.4. Photogrammetric Postural Analysis

Postural evaluation using photogrammetry has been previously demonstrated to be a reliable method in people with AIS [28,29]. In the present study, 4 digital photographs (front, back, right and left side) were recorded using a portable device (Tablet Huawei^®^ Mediapad, Shenzhen, Guangdong, China) in order to analyze sagittal and frontal planes. The device was set on a tripod, three meters away from the line marking the position of the participant. The height of the tripod was adjusted so the middle of the objective lens was 100 cm above the ground [30]. Each participant was positioned in front of the camera with a postural grid (ATS^®^, Arezzo, Italy) on the back [31]. The application APECS-AI Posture Evaluation and Correction System^®^ (New Body Technologies SAS, Grenoble, France) was used to evaluate absolute and relative angles in frontal and sagittal planes. Specifically, the anterior and posterior shoulders level, trunk position and waistline on the coronal plane were analyzed using Anterior Trunk Symmetry Index (ATSI) and Posterior Trunk Symmetry Index (POTSI) [7]. These indexes display asymmetries between the left and right sides of the body and represent an easy and rapid approach to investigate scoliotic people [32]. Both ATSI and POTSI consist of six sub-indexes: three measures of the vertical asymmetry—HDI (distances along the Y axis, between shoulder, axilla, and waist level) and three measures of the horizontal asymmetry—FAI (distances along the X axis, between C7/sternal notch, axilla, and waist level). To obtain ATSI and POTSI values, the following equations are used [6]:

ATSI = (FAI_SN + FAI_A + FAI_T) + (HDI_S + HDI_A + HDI_T)

POTSI = (FAI_C7 + FAI_A + FAI_T) + (HDI_S + HDI_A + HDI_T).

**Figure 2:**
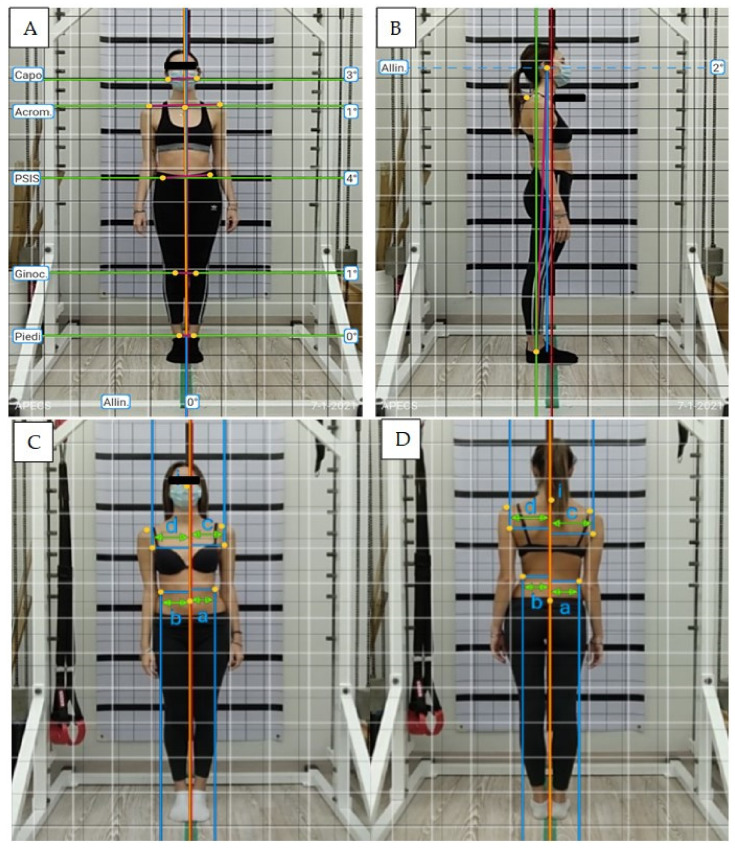
Four different photogrammetry analysis: (**A**) postural analysis on frontal plane, (**B**) postural analysis on sagittal plane, (**C**) ATSI evaluation, and (**D**) POTSI evaluation.

Figure 2 shows an example of photogrammetry analysis assessed by the APECS-AI application.

### 2.3. Statistical Analysis

The statistical analysis was performed with STATA^®^ software, version 17 (Publisher: StataCorp. 2021. Stata Statistical Software: Release 17. College Station, TX, USA, StataCorp LP). The Shapiro–Wilk test was assessed to check normality. Participants’ characteristics were reported as mean (±SD) and percentage of the observed feature. A multivariate mean test was performed (*Hotelling t*^2^ statistic) to analyze whether all the mean were the same; the *Hotelling F* and *p*-values were reported. The post hoc analysis using the two-tailed Student paired *t*-test was performed to compare each SRS-22 variable mean; the mean differences (95% C.I.), and *t* values were reported, the Bonferroni correction was calculated, and the *p*-value was settled at *α/m*, where *α* is the significance level and *m* is the number of possible comparisons. In addition, a comparison among domain three mean and the mean of all other domains was settled.

A pairwise correlation matrix with variables coefficients was calculated to analyze linear relationship. Then, the stepwise regression method was performed to describe the variability of SRS-22 domain three, explained by other variables. The backward elimination approach was used, which involved starting with all candidate variables, and the deletion of each variable was tested through the significant level as model fit criterion. Only variables with a significance level ≤0.1 and which explain at least the 7% of the response variable were included in the final model. The Adjusted R-squared was used to quantify the regression model’ goodness of fit. Additionally, the Fisher score was reported. Finally, four different linear regression models were performed to describe the variability of SRS-22 domain three, explained individually by TAPS, SpinalMouse, Photogrammetric postural analysis (ATSI and POTSI), and each SRS domain. The statistical significance level α was set at 0.05.

## 3. Results

Table 1 shows participants’ characteristics. Fifteen participants were recruited, with an age mean of 14.47 (±2.82) years old. The 31% was male and the 69% was female. The dorso-lumbar scoliosis (left or right) was the most common asymmetry observed (53.32%, equally distributed among left and right side).

Table 2 shows mean differences between female and male participants for each variable. Only ATSI means reported significant differences (*t*_13_ = 2.9, *p* = 0.01).

**Figure 3:**
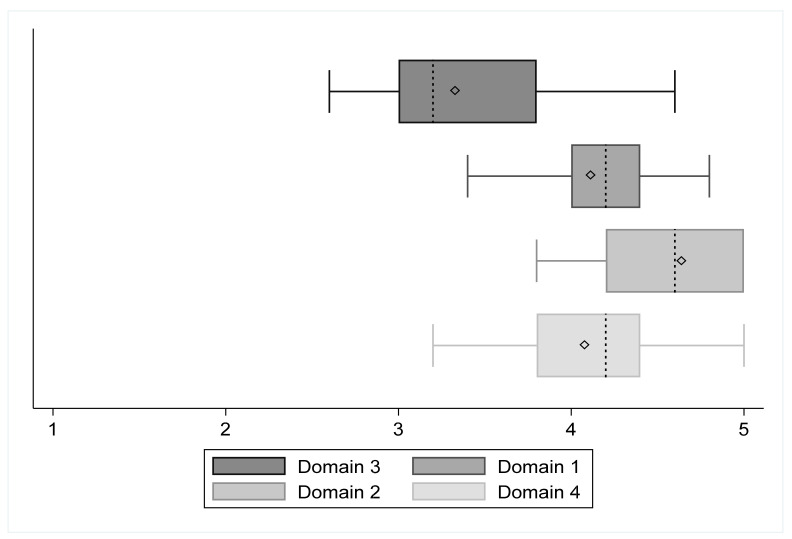
Boxplots of all SRS domains. Note: dash line represents median value, diamond represents mean value.

Figure 3 shows boxplots of all SRS domains. Each domain mean is displayed by a diamond within every box. Table 2 shows the summary statistics for multivariate mean differences analysis. *Hotelling t*^2^ = 70.29, *Hotelling F*_(3, 12)_ = 20.08, and the *p* < 0.001. The post hoc analysis included six comparisons, and the significance level was settled at 0.0083 (0.05/6). Each comparison among domain three (self-perceived body image) and other domains reported statistically significant differences: domains three-one [−0.84 (95% C.I.: −1.07, −0.61); *t* = −7.70; *p* < 0.001]; domains three-two [−1.27 (95% C.I.: −1.63, −0.90); *t* = −7.46; *p* < 0.001]; domains three-four [−0.81 (95% C.I.: −1.14, −0.49); *t* = −5.36; *p* < 0.001]. All other comparisons are available on Table 3. Only the difference between domain one (function/activity) and four (mental health) was not significant [−0.03 (95% C.I.: −0.34, 0.28); *t* = −0.18; *p* = 0.86].

**Figure 4:**
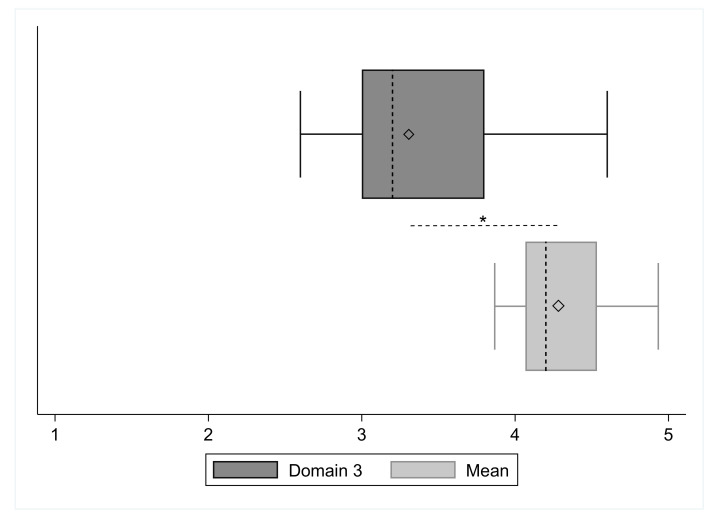
Boxplots of the SRS domain 3 and SRS mean of domains 1, 2 and 4 (Mean). The diamonds inside two boxes represent the respective mean. Note: *, *p* < 0.001.

Figure 4 shows boxplots of SRS domain three and the other domains mean (one, two and four), in which is displayed the relative mean difference (point distance among two diamonds). Its value equals to −0.97 (95% C.I.: −1.24; −0.70), the value of statistic *t* is −7.79, and *p* is <0.001.

Table 4 shows the coefficients correlation matrix in which all linear variable coefficients are reported. Totally twelve variables are included. The highest linear correlation coefficient value related to domain three is reported with the SRS domain one (*r* = 0.66), whereas the lowest is with SpinalMouse value on antero-posterior axis (*r* = 0.07).

Table 5 shows the stepwise regression model. At the beginning, seven predictor variables were selected (SpinalMouse on antero-posterior and lateral axis, and the curve degree; TAPS value; ATSI and POTSI; the mean of all SRS domains one, two and four). Although the model with five variables (with no POTSI and SpinalMouse on lateral axis) reported a discrete goodness of fit value (*R*^2^ = 0.67, *adjusted*-*R*^2^ = 0.49), only two variables (the mean of all SRS domains and the mean of TAPS) met the inclusion criteria, and the final model explained the 27.5% (*adjusted R*^2^) of the domain three variability (*p* = 0.05).

Finally, Table 6 displays each specific regression model for all instruments described [SRS-22 domains, TAPS, SpinalMouse^®^, Photogrammetry (ATSI and POTSI)]. The model that better explains the domain three variability is represented by SRS-22 domains, which describes the 42% (*adjusted R*^2^) of the response (D3). The better regressor seems to be the domain one (*t* = 3.24, *p* < 0.01). Differently, the photogrammetry model reports the lower goodness of fit value (*adjusted R*^2^ = 0.012). However, neither TAPS (*F*_(4,10)_ = 1.82; *p* = 0.2) or SpinalMouse (*F*_(4,10)_ = 1.09; *p* = 0.39) models report a significant value of Fisher statistic, and both exhibit low goodness of fit values (*adjusted R*^2^ = 0.05; *adjusted R*^2^ = 0.02).

## 4. Discussion

The main purpose of this study was to investigate how fifteen participants with diagnosis of mild AIS perceived their body image, and how the mild magnitude of the curve impacted their QoL. We found a low mean value of SRS-22 domain three, which suggested that spinal asymmetry negatively affects self-perceived body image, even if severity of scoliosis is less than 25°. To our knowledge, no previous studies mainly investigated this outcome in patients with mild curves using SRS-22. Several authors focused on discriminative validity of this tool about curve severity and QoL, but body perception was the main topic especially in moderate and severe scoliosis [20,32,33,34]. Normative data for SRS-22 have been reported in healthy people from different countries [35,36]. In these studies, SRS-22 domains were analyzed and compared about age, gender, anthropometric measurement, ethnicity, and familiar status. Self-image scores were significantly lower in Southeast Asian people compared to US and Ghana, with mean values of 3.9, 4.2 and 4.2, respectively (*p* < 0.0001) [35]. In this study, self-image domain scores decreased ranging from 4.25 in age 10 to 3.76 in age 17, evidencing a negative correlation (r = −0.166, *p* < 0.0001). Similarly, US people showed lower values in age 16–19 compared to 10–12, even if this difference was not significant (4.39 and 4.47, respectively). Berliner et al. evidenced good discriminative validity between small non-operative curves (<40° Cobb) and larger surgical curves (>40° Cobb) about pain, self-body image and SRS-22 total domain, with higher scores in non-operative curves compared to surgical curves [33]. In this study, self-image domain mean values were 4.1 for 0°–19° and 4.0 for 20–40° curves, significantly different from severe curves scores (3.5 for 41°–50° and 3.3 for 51°–60° curves, respectively). Wang et al. reported that self-image SRS-22 scores correlates negatively with main Cobb angle, apical vertebral translation, and razor hump height, independently from type of scoliosis (single or double curves and spine localization). These authors reported a mean value of 3.08 for a sample’s mean curvature of 45° Cobb. In our study, the mean SRS self-image domain score was 3.2, smaller than the results evidenced by Berliner et al. for mild curves. Contrarily, the mean values for other SRS-22 domains in our sample were similar to previous authors (higher than 4.0). Furthermore, our result is close to SRS-22 self-image score reported by Makino [37]. In this research, the authors investigated QoL and low back pain (LBP) in 111 female patients with nonsurgical scoliosis (mean Cobb angle range: 27.5°–36.1°). The SRS-22 self-image scores were 2.8 and 3.0 for LBP and No Pain groups, while other domains evidenced higher values (greater than 3.8 and 4.3 for LBP and No Pain groups, respectively). According to these findings, many studies underline that as greater is the curve magnitude as worser is the body-image perception [13,19,20,32]. Although in our study the participants Cobb angle was less than 25°, the results report a negative self-image perception and suggest the importance of proper assessment in mild scoliosis too. Despite SRS-22 have been commonly used for patient-outcome analysis, other specific questionnaire as the BIDQ-S (Body Image Disturbance Questionnaire-Scoliosis) could be selected [38]. This tool was recently validated to measure the body image perception in AIS people and showed a strong correlation with SRS total score [39]. In addition, Cheshire et al. reported that SRS-22 self-image domain has a weak strength of correlation with external measure of spine deformity (ISIS2 surface topography) and suggested the use of pictorial scale like TAPS [34]. For this reason, we integrated SRS-22 with TAPS assessment in order to better understand body perception. However, the small sample size and the absence of a comparison group with Cobb angle greater than 25° must be considered as a limit.

The second intend of the current research was to analyze how participants with mild AIS perceived their quality of life and which SRS-22 domain could mostly influence the results. According with our expectations, domain three reported the lowest value when compared with each other domain and the total mean of the other domains, which suggests that the body-image perception could be the most critical factor that negatively affects the adolescent QoL when scoliosis is not severe. In agreement with this result, some researchers observed a significant linear correlation between poorer body image perception and poorer QoL, regardless of the Cobb angle magnitude [13]. Additionally, Payne and colleagues found that adolescent with idiopathic scoliosis are more likely to be dissatisfied with their appearance than adolescents with no scoliosis, and this condition could impact their psychosocial growth [40]. Consequently, greater relevance should be attributed to the body-image progression in adolescent with mild scoliosis and its effect on their quality of life. Recently, Kinel et al. suggested the use of ISYQOL (Italian Youth Quality of Life Questionnaire) for mild and moderate scoliosis [41]. In this research, authors evidenced higher metric properties for ISYQOL compared to SRS-22 in 81 female patients with mean Cobb angle of 31°. In particular, the severity of scoliosis (10°–30° vs. >30°) demonstrated a direct statistically significant effect on QoL when evaluated with ISYQOL only. However, since ISYQOL is a unidimensional scale, it could not be possible to analyze which domain mostly impacts QoL as performed in SRS-22. The absence of a follow-up to evaluate the health-related quality of life progression and the use of SRS-22 alone must be considered. In addition, we could not draw conclusions on psychological factors due to the absence of psycho-social measurements.

Interestingly, we found that the SRS-22 perceived pain domain reported the highest mean value, which indicated that participants did not suffer of severe pain. The mean score presented in this study and the values evidenced by Berliner et al. for mild and moderate scoliosis are the same (SRS-22 pain domain: 4.6) [33]. Our result agrees with authors who suggested that mild scoliosis rarely causes pain and impairments in adolescent, and recognized AIS as a spinal deformity without pain [42,43]. Despite several studies found that patients with AIS experienced pain more than control subjects with no scoliosis, further comparisons are needed among people with different curve magnitude (mild, moderate, and severe) [44,45]. However, many factors, such as radiographic parameters, psychological and mental distress, dissatisfaction, social influences, and hormonal status, could affect the perceived pain, and further investigations in this direction were beyond our aims.

The final purpose was to analyze whether the variability of the self-perceived body image could be explained by other SRS-22 domains (function/activity, pain, and mental health), self-perception of the trunk measured by TAPS, curvature and mobility of the spinal column in the frontal and sagittal planes evaluated by SpinalMouse^®^, and the anterior and posterior asymmetries between left and right sides of the trunk measured by photogrammetry (ATSI, POTSI). To the authors’ knowledge, no previous study speculated on these relations and few comparisons were possible. However, our results show that the SRS-22’ domains one, two and four are good predictors of the domain three variability, explaining about 55% of it. Since health-related quality of life must be strongly considered for successful treatment of spinal deformities, we suppose that monitoring the QoL in people with mild idiopathic scoliosis is a primary necessity to prevent altered self-image perception during growth [21,41]. Further analysis and comparisons between specific QoL questionnaire and the SRS-22 are requested to correctly manage this severity of curvature too.

About TAPS, one study agrees with our results and reports a discrete linear correlation between the domain three and this pictorial scale (*r* = 0.46), but no regression model was assessed [22].

Differently, worse models are described by SpinalMouse^®^ and photogrammetry. These results suggest that the curvature and mobility of the spine and trunk asymmetries do not allow us to predict the self-image perception in our sample. Previously, Brewer et al. reported that volumetric asymmetries parameters measured by surface topography correlate better than Cobb angle with body perception and mental health, while Matamalas et al. demonstrated significant correlation between waistline asymmetry and body perception of deformity [46,47]. In addition, some questions about the role of shoulder balance using photogrammetry in trunk assessment for non-operated scoliosis have been posed [47]. In this research, the authors underlined the importance to integrate surface topography with additional parameters like shoulder balance and waistline assessment. We suppose that low Cobb angle value, torso shape and poor trunk deformity evaluated by ATSI and POTSI could not correctly predict self-perceived body imagine in mild scoliosis. Despite this, the integration between specific QoL questionnaire (TAPS and SRS-22) and proper measurement (like photogrammetry and Spinal Mouse^®^) is recommended to monitor scoliosis progression in non-invasive, quick, and scientific way.

The sample size, participants age and psychological characteristics, differences about type of curvature and phase of treatment represent a limit in our work. Future investigations are needed about the role of each evaluation tool in mild AIS compared with greater curvatures, in relation to specific scoliosis approach.

## 5. Conclusions

The mild magnitude of the spinal curve negatively affects self-perceived body image measured by SRS-22 in people with AIS. Additionally, SRS-22 self-image domain impacts on SRS-22 total score and it evidences significant differences with other domains in people with mild scoliosis. The use of this questionnaire should be integrated with pictorial scale (TAPS) to better understand the role of body perception in patient-reported quality of life. Despite the absence of correlation with self-image perception in spinal curvatures smaller than 25°, Spinal Mouse^®^ and photogrammetry are useful and non-invasive tool that can correctly monitor scoliosis evolution and highlight treatment progression.

## Figures and Tables

**Table 1:** Participants’ characteristics.

Subject	Age	Gender	Scoliosis
1	15	female	dorso-lumbar left
2	14	female	dorso-lumbar right
3	13	female	dorso-lumbar left
4	12	female	dorsal left
5	13	male	dorsal right
6	22	female	dorso-lumbar right
7	15	male	dorso-lumbar left
8	13	female	lumbar left
9	12	female	dorso-lumbar left
10	13	male	dorso-lumbar right
11	12	male	lumbar left
12	18	female	dorso-lumbar right
13	16	male	dorsal right
14	12	female	dorsal right
15	17	female	lumbar left
	average = 14.47 (±2.82)	male = 31%	dorso-lumbar left = 26.66%
		female = 69%	dorso-lumbar right = 26.66%
			lumbar left = 20%
			dorsal right = 20%
			dorsal left = 6.7%

**Table 2:** Variables statistics by gender.

Variable	Total (n = 15)	Female (n = 10)	Male (n = 5)	Δ		
	Mean (±SD)	Mean (±SD)	Mean (±SD)	Mean [95% C. I.]	*t*_(13)_	*p*
Age	14.47 (±2.82)	14.8 (±3.29)	13.8 (±1.64)	1 [−2.42, 4.42]	0.63	0.54
SM antero/posterior	0.8 (±3.53)	−0.3 (±3.83)	3 (±1.22)	−3.3 [−7.16, 0.56]	−1.85	0.08
SM lateral	−0.6 (±2.2)	−0.1 (±2.13)	−1.6 (±2.2)	1.5 [−1.04, 4.04]	1.27	0.22
SM curvature	8.2 (±5.3)	8.7 (±5.81)	7.2 (±4.44)	1.5 [−4.92, 7.92]	0.5	0.62
ATSI	16.73 (±10.7)	21.3 (±10.1)	7.6 (±3.65)	13.7 [3.47, 23.93]	2.9	0.01 *
POTSI	18.8 (±6.65)	16.9 (±4.77)	22.6 (±8.73)	−5.7 [−13.11, 1.71]	−1.66	0.12
TAPS	3.6 (±0.40)	3.63 (±0.46)	3.53 (±0.3)	0.1 [−0.39, 0.59]	0.44	0.67
SRS domain 1	4.15 (±0.37)	4.14 (±0.42)	4.16 (±0.26)	−0.02 [−0.47, 0.43]	−0.1	0.92
SRS domain 2	4.57 (±0.41)	4.62 (±0.43)	4.48 (±0.41)	0.14 [−0.36, 0.64]	0.6	0.56
SRS domain 3	3.30 (±0.56)	3.26 (±0.57)	3.4 (±0.58)	−0.14 [−0.82, 0.54]	−0.44	0.66
SRS domain 4	4.12 (±0.44)	4.12 (±0.51)	4.12 (±0.30)	0 [−0.54, 0.54]	0	0.99
SRS total mean	4.28 (±0.28)	4.29 (±0.33)	4.25 (±0.20)	0.04 [−0.31, 0.39]	0.25	0.81

Note: n, number of observations; C. I., confidence interval; SM, SpinalMouse; SD, standard deviation; ∆, differences among gender; *t*, Student test; *, statistical significance.

**Table 3:** Post hoc summary statistics.

Domains	Mean Diff	95% C.I.		*t*	*p*
D3-D1	−0.84	−1.07	−0.61	−7.70	<0.001 *
D3-D2	−1.26	−1.63	−0.90	−7.46	<0.001 *
D3-D4	−0.81	−1.14	−0.49	−5.36	<0.001 *
D2-D1	0.42	0.14	0.71	3.26	0.005 *
D2-D4	0.45	0.21	0.70	4.01	0.001 *
D4-D1	−0.02	−0.34	0.28	−0.18	0.85

Note: *, statistical significance.

**Table 4:** Coefficients correlation matrix.

	SpinM A/P	SpinM L	SpinM C	ATSI	POTSI	TAPS M	D1	D2	D3	D4	SRSMean
SpinM A/P	1										
SpinM L	−0.08	1									
SpinM C	−0.34	−0.39	1								
ATSI	−0.76	0.52	0.08	1							
POTSI	0.21	−0.46	−0.07	−0.34	1						
TAPS M	0.34	0.09	−0.23	−0.20	0.30	1					
D1	0.29	−0.15	−0.40	−0.24	0.23	0.13	1				
D2	−0.01	0.11	0.12	0.05	0.02	−0.33	0.16	1			
D3	0.07	−0.16	−0.33	−0.25	0.37	0.35	0.66	0.11	1		
D4	0.27	0.23	−0.11	−0.2	0.07	0.18	0.04	0.47	0.33	1	
SRSMean	0.26	0.10	−0.17	−0.18	0.15	−0.01	0.5	0.79	0.50	0.76	1

Note: SpinM A/P, SpinalMouse anterior-posterior; SpinM L, SpinalMouse Lateral; SpinM C, SpinalMouse Curves degree; TAPS M, mean of summary of all TAPS figures; D1, mean of SRS-22 domain 1; D2, mean of SRS-22 domain 2; D3, mean of SRS-22 domain 3; D4, mean of SRS-22 domain 4; D5, mean of SRS-22 domain 5; SRSMean, the mean of all SRS domains 1, 2 and 4.

**Table 5:** Stepwise regression analysis: the backward procedure.

Source	SS	df	MS			
Model	1.66	1	0.83		obs	15
Residual	2.73	13	0.23		*F*_(1,13)_	3.65
Total	4.39	14	0.31		Prob > F	0.05
					*R* ^2^	0.38
					*Adj R* ^2^	0.27
					Root MSE	0.48
D3	Β	S.E.	*t*	*p*	95% C.I.	
Intercept	−2.71	2.23	−1.21	0.25	−7.58	2.16
TAPSM	0.49	0.32	1.55	0.15	−0.2	1.18
SRSmean	0.99	0.45	2.22	0.05	0.02	1.96

Note: D3, domain 3; obs, number of observations; SRSmean, mean of all SRS domains with no domain 3 and 5; TAPSM, mean of TAPS Figure 1, Figure 2 and Figure 3; S.E., standard error.

**Table 6:** Regression models.

Source	SS	df	MS			
Model	2.39	3	0.8		obs	15
Residual	2	11	0.11		*F*_(4,10)_	4.39
Total	4.39	14	0.31		Prob > F	0.03
					*R* ^2^	0.54
					*Adj R* ^2^	0.42
					Root MSE	0.43
D3	Β	S.E.	*t*	*p*	95% C.I.	
Intercept	−1.85	1.77	−1.04	0.32	−5.75	2.06
D1	1.02	0.32	3.24	0.01	0.33	1.72
D2	−0.24	0.32	−0.76	0.47	−0.94	0.46
D4	0.49	0.29	1.66	0.13	−0.16	1.14
**TAPS**					obs	15
Source	SS	df	MS		*F*_(4,10)_	1.82
Model	0.54	1	0.54		Prob > *F*	0.2
Residual	3.85	13	0.3		*R* ^2^	0.12
Total	4.39	14	0.31		*Adj* *R* ^2^	0.05
					Root MSE	0.54
D3	Β	S.E.	*t*	*p*	95% C.I.	
Intercept	1.55	1.31	1.18	0.26	−1.28	4.38
TAPSM	0.49	0.36	1.35	0.2	−0.29	1.27
**SpinalMouse**					obs	15
Source	SS	df	MS		*F*_(4,10)_	1.09
Model	1.00	3	0.33		Prob > *F*	0.39
Residual	3.39	11	0.31		*R* ^2^	0.23
Total	4.39	14	0.31		*Adj* *R* ^2^	0.02
					Root MSE	0.55
D3	Β	S.E.	*t*	*p*	95% C.I.	
Intercept	3.73	0.31	12.08	<0.001	3.05	4.40
SpinM A/P	−0.02	0.05	−0.49	0.63	−0.12	0.08
SpinM L	−0.1	0.07	−1.29	0.22	−0.26	0.07
SpinM C	−0.06	0.03	−1.68	0.12	−0.13	0.02
**Photogrammetry**					obs	15
Source	SS	df	MS		*F*_(4,10)_	1.09
Model	0.67	2	0.34		Prob > *F*	0.37
Residual	3.72	12	0.31		R2	0.15
Total	4.39	4	0.31		Adj R2	0.01
					Root MSE	0.56
D3	β	S.E.	*t*	*p*	95% C.I.	
Intercept	2.93	0.6	4.9	<0.001	1.63	4.23
ATSI	−0.01	0.01	−0.51	0.62	−0.04	0.02
POTSI	0.03	0.02	1.13	0.28	−0.02	0.09

## Data Availability

Data have been shared via OSF.io (https://osf.io/wcq2h/?view_only=a7c16c3f100040cf86ce00e70a6c03d7, accessed on 28 February 2022.

## References

[1] Negrini S., Donzelli S., Aulisa A.G., Czaprowski D., Schreiber S., De Mauroy J.C., Diers H., Grivas T.B., Knott P., Kotwicki T. (2018). 2016 SOSORT guidelines: Orthopaedic and rehabilitation treatment of idiopathic scoliosis during growth. Scoliosis Spinal Disord..

[2] De Baat P. (2012). Scoliose: Overzicht van typen, oorzaken, diagnostiek en behandeling 1. [Scoliosis: Review of types, aetiology, diagnostics, and treatment 1]. Ned. Tijdschr. Voor Tandheelkd..

[3] De Baat P., van Biezen F.C., de Baat C. (2012). Scoliose: Overzicht van typen, oorzaken, diagnostiek en behandeling 2. [Scoliosis: Review of types, aetiology, diagnostics, and treatment 2]. Ned. Tijdschr. Voor Tandheelkd..

[4] Ueno M., Takaso M., Nakazawa T., Imura T., Saito W., Shintani R., Uchida K., Fukuda M., Takahashi K., Ohtori S. (2011). A 5-year epidemiological study on the prevalence rate of idiopathic scoliosis in Tokyo: School screening of more than 250,000 children. J. Orthop. Sci..

[5] Weinstein S.L., Dolan L., Cheng J., Danielsson A., Morcuende J.A. (2008). Adolescent idiopathic scoliosis. Lancet.

[6] Patias P., Grivas T.B., Kaspiris A., Aggouris C., Drakoutos E. (2010). A review of the trunk surface metrics used as Scoliosis and other deformities evaluation indices. Scoliosis.

[7] Stolinski L., Kozinoga M., Czaprowski D., Tyrakowski M., Cerny P., Suzuki N., Kotwicki T. (2017). Two-dimensional digital photography for child body posture evaluation: Standardized technique, reliable parameters and normative data for age 7–10 years. Scoliosis Spinal Disord..

[8] Livanelioglu A., Kaya F., Nabiyev V., Demirkıran G., Firat T., Demirkiran G., Fırat T. (2015). The validity and reliability of “Spinal Mouse” assessment of spinal curvatures in the frontal plane in pediatric adolescent idiopathic thoraco-lumbar curves. Eur. Spine J..

[9] Leal J.S., Aroeira R.M.C., Gressler V., Greco M., Pertence A.E.M., Lamounier J.A. (2019). Accuracy of photogrammetry for detecting adolescent idiopathic scoliosis progression. Spine J..

[10] Bettany-Saltikov J., Weiss H.-R., Chockalingam N., Taranu R., Srinivas S., Hogg J., Whittaker V., Kalyan R.V., Arnell T. (2015). Surgical versus non-surgical interventions in people with adolescent idiopathic scoliosis. Cochrane Database Syst. Rev..

[11] Weiss H.-R., Goodall D. (2008). The treatment of adolescent idiopathic scoliosis (AIS) according to present evidence. A systematic review. Eur. J. Phys. Rehabil. Med..

[12] Ascani E., Bartolozzi P., Logroscino C.A., Marchetti P.G., Ponte A., Savini R., Travaglini F., Binazzi R., DI Silvestre M. (1986). Natural History of Untreated Idiopathic Scoliosis After Skeletal Maturity. Spine.

[13] Schwieger T., Campo S., Weinstein S.L., Dolan L.A., Ashida S., Steuber K.R. (2016). Body Image and Quality-of-Life in Untreated Versus Brace-Treated Females with Adolescent Idiopathic Scoliosis. Spine.

[14] Essex R., Bruce G., Dibley M., Newton P., Thompson T., Swaine I., Dibley L. (2022). A systematic scoping review and textual narrative synthesis of the qualitative evidence related to adolescent idiopathic scoliosis. Int. J. Orthop. Trauma Nurs..

[15] Gallant J.-N., Morgan C.D., Stoklosa J.B., Gannon S.R., Shannon C.N., Bonfield C.M. (2018). Psychosocial Difficulties in Adolescent Idiopathic Scoliosis: Body Image, Eating Behaviors, and Mood Disorders. World Neurosurg..

[16] Monticone M., Carabalona R., Negrini S. (2004). Reliability of the Scoliosis Research Society-22 Patient questionnaire (Italian Version) in mild adolescent vertebral deformities. Eur. Med. Phys..

[17] Matamalas A., D’Agata E., Sanchez-Raya J., Bago J. (2016). Trunk appearance perception scale for physicians (TAPS-Phy)-A valid and reliable tool to rate trunk deformity in idiopathic scoliosis. Scoliosis Spinal Disord..

[18] Asher M., Lai S.M., Burton D., Manna B. (2002). Spine Deformity Correlates Better Than Trunk Deformity with Idiopathic Scoliosis Patients’ Quality of Life Questionnaire Responses. Stud. Health Technol. Inform..

[19] Parent E.C., Hill D., Mahood J., Moreau M., Raso J., Lou E. (2009). Discriminative and predictive validity of the scoliosis research society-22 questionnaire in management and curve-severity subgroups of adolescents with Idiopathic Scoliosis. Spine.

[20] Wang L., Wang Y., Yu B., Zhang J., Shen J., Qiu G., Li Y. (2014). Relation between self-image score of SRS-22 with deformity measures in female adolescent idiopathic scoliosis patients. Orthop. Traumatol. Surg. Res..

[21] Caronni A., Donzelli S., Zaina F., Negrini S. (2019). The Italian Spine Youth Quality of Life questionnaire measures health-related quality of life of adolescents with spinal deformities better than the reference standard, the Scoliosis Research Society 22 questionnaire. Clin. Rehabil..

[22] Matamalas A., Bagó J., D’Agata E., Pellisé F. (2014). Body image in idiopathic scoliosis: A comparison study of psychometric properties between four patient-reported outcome instruments. Health Qual. Life Outcomes.

[23] Savvides P., Gerdhem P., Grauers A., Danielsson A., Diarbakerli E. (2020). Self-Experienced Trunk Appearance in Individuals with and Without Idiopathic Scoliosis. Spine.

[24] Asher M., Lai S.M., Burton D., Manna B. (2003). The Reliability and Concurrent Validity of the Scoliosis Research Society-22 Patient Questionnaire for Idiopathic Scoliosis. Spine.

[25] Bago J., Sánchez-Raya J., Perez-Grueso F.J.S., Climent J.M. (2010). The Trunk Appearance Perception Scale (TAPS): A new tool to evaluate subjective impression of trunk deformity in patients with idiopathic scoliosis. Scoliosis.

[26] Mannion A.F., Knecht K., Balaban G., Dvorak J., Grob D. (2004). A new skin-surface device for measuring the curvature and global and segmental ranges of motion of the spine: Reliability of measurements and comparison with data reviewed from the literature. Eur. Spine J..

[27] Post R.B., Leferink V.J.M. (2004). Spinal mobility: Sagittal range of motion measured with the SpinalMouse, a new non-invasive device. Arch. Orthop. Trauma. Surg..

[28] Ruggeri S.K., Siqueira C.A., Ribeiro A.P., Amado João S.M. (2012). Reliability of photogrammetry in the evaluation the postural aspects of individuals with structural scoliosis. J. Bodyw. Mov. Ther..

[29] Penha P.J., Penha N.L.J., De Carvalho B.K.G., Andrade R.M., Schmitt A.C.B., João S.M.A. (2017). Posture Alignment of Adolescent Idiopathic Scoliosis: Photogrammetry in Scoliosis School Screening. J. Manip. Physiol. Ther..

[30] Porto A.B., Okazaki V.U.A. (2017). Procedures of assessment on the quantification of thoracic kyphosis and lumbar lordosis by radiography and photogrammetry: A literature Review. J. Bodyw. Mov. Ther..

[31] Russo L. (2019). Biomeccanica: Principi di Biomeccanica e applicazioni della video analisi al movimento umano. ATS Giacomo Catalani Ed..

[32] Asher M., Lai S.M., Burton D., Manna B. (2003). Discrimination Validity of the Scoliosis Research Society-22 Patient Questionnaire. Spine.

[33] Berliner J.L., Verma K., Lonner B.S., Penn P.U., Bharucha N.J. (2013). Discriminative validity of the Scoliosis Research Society 22 questionnaire among five curve-severity subgroups of adolescents with idiopathic scoliosis. Spine.

[34] Cheshire J., Gardner A., Berryman F., Pynsent P. (2017). Do the SRS-22 self-image and mental health domain scores reflect the degree of asymmetry of the back in adolescent idiopathic scoliosis?. Scoliosis Spinal Disord..

[35] Verma K., Nathan S.T., Comer C.D., Lonner B., Shah S.A. (2017). A Normative Baseline for the Srs-22 From Over 1000 Healthy Adolescents in India: Which Demographic Factors Affect Outcome?. Spine.

[36] Daubs M.D., Hung M., Neese A., Hon S.D., Lawrence B.D., Patel A.A., Annis P., Smith J., Brodke D.S. (2014). Scoliosis Research Society-22 Results in 3052 Healthy Adolescents Aged 10 to 19 Years. Spine.

[37] Makino T., Kaito T., Kashii M., Iwasaki M., Yoshikawa H. (2015). Low back pain and patient-reported QOL outcomes in patients with adolescent idiopathic scoliosis without corrective surgery. Springerplus.

[38] Auerbach J.D., Lonner B.S., Crerand C.E., Shah S.A., Flynn J.M., Bastrom T., Penn P., Ahn J., Toombs C., Bharucha N. (2014). Body image in patients with adolescent idiopathic scoliosis: Validation of the Body Image Disturbance Questionnaire--Scoliosis Version. J. Bone Joint Surg. Am..

[39] Bauer J.M. (2021). The body image disturbance questionnaire-scoliosis better correlates to quality of life measurements than the spinal assessment questionnaire in pediatric idiopathic scoliosis. Spine Deform..

[40] Payne W.K. (1997). III.; Ogilvie, J.W.; Resnick, M.D.; Kane, R.L.; Transfeldt, E.E.; Blum, R.W. Does scoliosis have a psychological impact and does gender make a difference?. Spine.

[41] Kinel E., Korbel K., Kozinoga M., Czaprowski D., Stępniak Ł., Kotwicki T. (2021). The Measurement of Health-Related Quality of Life of Girls with Mild to Moderate Idiopathic Scoliosis—Comparison of ISYQOL versus SRS-22 Questionnaire. J. Clin. Med..

[42] Aebi M. (2005). The adult scoliosis. Eur. Spine J..

[43] Kuznia A.L., Hernandez A.K., Lee L.U. (2020). Adolescent Idiopathic Scoliosis: Common Questions and Answers. Am. Fam. Physician.

[44] Mayo N.E., Goldberg M.S., Poitras B., Scott S., Hanley J. (1994). The Ste-Justine Adolescent Idiopathic Scoliosis Cohort Study. Spine.

[45] Sato T., Hirano T., Ito T., Morita O., Kikuchi R., Endo N., Tanabe N. (2010). Back pain in adolescents with idiopathic scoliosis: Epidemiological study for 43,630 pupils in Niigata City, Japan. Eur. Spine J..

[46] Brewer P., Berryman F., Baker D., Pynsent P.B., Gardner A. (2013). Influence of Cobb Angle and ISIS2 Surface Topography Volumetric Asymmetry on Scoliosis Research Society-22 Outcome Scores in Scoliosis. Spine Deform..

[47] Matamalas A., Bagó J., D’agata E., Pellisé F. (2016). Validity and reliability of photographic measures to evaluate waistline asymmetry in idiopathic scoliosis. Eur. Spine J..

